# Imidacloprid-Induced Impairment of Mushroom Bodies and Behavior of the Native Stingless Bee *Melipona quadrifasciata anthidioides*

**DOI:** 10.1371/journal.pone.0038406

**Published:** 2012-06-04

**Authors:** Hudson Vaner V. Tomé, Gustavo F. Martins, Maria Augusta P. Lima, Lúcio Antonio O. Campos, Raul Narciso C. Guedes

**Affiliations:** 1 Departamento de Entomologia, Universidade Federal de Viçosa, Viçosa, Minas Gerais, Brazil; 2 Departamento de Biologia Geral, Universidade Federal de Viçosa, Viçosa, Minas Gerais, Brazil; 3 Departamento de Biologia Animal, Universidade Federal de Viçosa, Viçosa, Minas Gerais, Brazil; University of Kentucky, United States of America

## Abstract

Declines in pollinator colonies represent a worldwide concern. The widespread use of agricultural pesticides is recognized as a potential cause of these declines. Previous studies have examined the effects of neonicotinoid insecticides such as imidacloprid on pollinator colonies, but these investigations have mainly focused on adult honey bees. Native stingless bees (Hymenoptera: Apidae: Meliponinae) are key pollinators in neotropical areas and are threatened with extinction due to deforestation and pesticide use. Few studies have directly investigated the effects of pesticides on these pollinators. Furthermore, the existing impact studies did not address the issue of larval ingestion of contaminated pollen and nectar, which could potentially have dire consequences for the colony. Here, we assessed the effects of imidacloprid ingestion by stingless bee larvae on their survival, development, neuromorphology and adult walking behavior. Increasing doses of imidacloprid were added to the diet provided to individual worker larvae of the stingless bee *Melipona quadrifasciata anthidioides* throughout their development. Survival rates above 50% were only observed at insecticide doses lower than 0.0056 µg active ingredient (a.i.)/bee. No sublethal effect on body mass or developmental time was observed in the surviving insects, but the pesticide treatment negatively affected the development of mushroom bodies in the brain and impaired the walking behavior of newly emerged adult workers. Therefore, stingless bee larvae are particularly susceptible to imidacloprid, as it caused both high mortality and sublethal effects that impaired brain development and compromised mobility at the young adult stage. These findings demonstrate the lethal effects of imidacloprid on native stingless bees and provide evidence of novel serious sublethal effects that may compromise colony survival. The ecological and economic importance of neotropical stingless bees as pollinators, their susceptibility to insecticides and the vulnerability of their larvae to insecticide exposure emphasize the importance of studying these species.

## Introduction

Honey bee populations (*Apis mellifera* L.) have been drastically declining for the last 60 years despite the widespread recognition of their importance as plant pollinators throughout the world [1]. Their decline is broadly attributed to the poorly understood phenomenon of Colony Collapse Disorder (CCD) [2], [3]. The rapid loss of adult bees, but not queens or brood, within a colony compromises colony defense against robber bees and other arthropod pests and is one of the main symptoms of CCD-affected colonies [2], [4]. The lack of dead adult bees within and around the affected hives suggests that they most likely die while foraging [5].

Many factors affect managed bee colonies, including diseases, parasites and pesticides [6]. Although no consensus has emerged on the main causes of colony decline, the multifactorial hypothesis has received recent support [7]. Nevertheless, pesticide exposure is a potential cause of bee colony loss in Europe and the United States, with the neonicotinoid insecticides serving as the main focus of concern [8]–[10]. This group of insecticides acts as agonists of (nicotinic) acetylcholine receptors (nAChR), leading to persistent activation of cholinergic synapses, hyperexcitation and eventual death [11], [12].

Imidacloprid was the first neonicotinoid to be marketed. This pesticide exhibits a broad spectrum of activity, plant translocation and persistence as well as application versatility, leading it to become one of the best-selling pesticides in the world [12], [13]. Imidacloprid residues can accumulate in pollen, nectar and wax, incurring a high risk to bees [9], [14], [15]. Furthermore, as a systemic compound (i.e., transported within the plant via the xylem), imidacloprid can even reach the leaves through guttation when applied to seeds, revealing yet another route by which bees can be exposed to this compound [16]. In addition, chromatography and mass spectrometry techniques detected lethal and sublethal concentrations of imidacloprid in the sap of plants originating from treated seeds [17], [18]. Several European countries, including France and Italy, suspended imidacloprid seed treatments, considering its residues to be the main factor responsible for bee population declines [9], [19], [20]. In contrast, imidacloprid use is extensive in tropical areas, particularly in Brazilian agricultural fields [21]. Unfortunately, the risk of exposure and impacts on native bee species have not been carefully addressed [22], [23].

Assessments of pesticide impacts on non-target species, including bees, rely heavily on acute toxicity bioassays [24]. Recently, however, alternative methods with greater potential for determining sublethal toxic effects on non-target species have been adopted [25]. The most frequent sublethal effects of pesticides on the honey bee include learning impairment [26], [27], memory reduction [28]–[30] and abnormal foraging behavior [31]–[33]. Several bee behavioral traits, especially foraging behavior, greatly depend on learning and memory. These activities are controlled by specific regions of the brain, which have consequently been a focus of studies on insecticide exposure [29], [34]. One such region is the mushroom body, where information gathered inside and outside the colony is stored. This structure expands with age and exhibits high neural plasticity during the adult stage [35]–[38]. Bees consuming low amounts of insecticide via either contaminated nectar or pollen can lose their cognitive abilities, leading to behavioral changes [26], [31], [32], [39]. The loss of adult bees is potentially harmful to the colony, but pesticide-induced changes that occur during larval development might have additional consequences for the colony and should not be neglected, particularly in pesticide impact studies [25].

Suitable methods for exposing larvae to pesticides have recently been developed and are pivotal for risk assessment studies in bee populations [40]–[44]. Unfortunately, these studies have mostly focused on the honey bee; very important pollinators in tropical regions, including stingless bee species (Hymenoptera: Apidae: Meliponinae), are seldom considered in these studies, despite their ecological and physiological differences from the honey bee [45]–[48]. Some stingless bee species are threatened with extinction in Brazil, with intensive pesticide use considered one of the main causes [45], [49]–[51]. Larval exposure to insecticides has yet to be studied in stingless bees, despite the high risk of exposure via the presence of contaminated pollen and nectar in larval diets and the potentially dire consequences for host colonies.

Here, we report a method for exposing stingless bee larvae to insecticides and assess the survival, development and behavior of stingless bee workers of the species *Melipona quadrifasciata anthidioides* Lepeletier exposed to imidacloprid via a contaminated diet during larval development. Our results indicate that stingless bee larvae are highly susceptible to imidacloprid. When applied at sublethal doses, the pesticide caused neuromorphological changes in the mushroom bodies and impaired walking behavior in young adults not yet able to fly. The implications of these findings for the structure, organization and survival of stingless bee colonies are discussed.

## Materials and Methods

### Ethics Statement

No specific permits were required for the described studies, which were carried out in the laboratory without depleting the original colonies from which the eggs were obtained. The insect colonies were initially established from hives obtained within the campus and maintained at the Experimental Apiary of the Federal University of Viçosa. Although some native stingless bees are considered endangered species in Brazil, including *Melipona capixaba* Moure & Camargo, the species here studied – *Melipona quadrifasciata anthidioides* is not an endangered or protected species.

### Stingless Bee Colonies

Five colonies of *M. quadrifasciata anthidioides* were collected in Viçosa county (MG, Brazil; 20° 45′ S and 42° 52′ W) and maintained at the Experimental Apiary at the Federal University of Viçosa for use in bioassays. Brood chambers containing eggs were removed from the hives and transferred to artificial cells containing 130 µL diet (added with 10 µL water), which provided sufficient sustenance for the full span of larval development. The artificial cells were made with honey bee wax and placed in the wells of polyethylene microplates (24-well plates with round-bottom wells). Each larval cell was maintained in a microplate well covered with a circular (honey bee) wax cap. The larval diet was collected from the same hives as the larvae. The artificial brood chambers were maintained at 28±1°C, 95±5% relative humidity (r.h.) and 24 h scotophase until the end of the feeding period. The artificial brood chambers were removed at the end of the larval period and transferred to new artificial brood chambers maintained at 28±1°C, 70±10% r.h. and 24 h, similar to the natural conditions.

### Insecticide

The insecticide used in this study was the neonicotinoid imidacloprid (water-dispersible granules at 700 g active ingredient (a.i.)/L; Bayer CropScience, São Paulo, Brazil). Water (distilled and deionized) was used as a carrier for the commercial insecticide formulation, which was applied at the following doses: 0.0, 0.0056, 0.014, 0.028, 0.037, 0.051, 0.056, 0.08, 0.112, 0.28, 0.37, 0.56, 1.12, 1.75, 3.50, 7.00, 14.00, 28.00 or 56 µg a.i./bee. The highest concentration corresponded to the commercial label rate (translated into the dose 56 µg a.i./bee according to the local application conditions) registered at the Brazilian Ministry of Agriculture for controlling the white fly (*Bemisia tabaci* (Gennadius) (Sternorrhyncha: Aleyrodidae) in tomato fields [52]. This crop is frequently treated with imidacloprid and relies on the stingless bee species *M. quadrifasciata anthidioides* as an important pollinator [46]–[48].

### Rearing Stingless Bees and Imidacloprid Bioassays

The larvae were maintained as described above. Upon emergence, the adult workers were marked with atoxic paint of different colors (Brasilux®, São Paulo, SP, Brazil) to facilitate age monitoring. The newly emerged adult workers were maintained in glass-covered wooden boxes (12×12×3 cm) placed within rearing chambers and fed with honey and pollen syrup. The young adult worker bees were collected for neuromorphological and behavioral analysis at one, four and eight days after emergence.

The stingless bee larvae were exposed to imidacloprid via their diet. The compound was mixed into the 10 µL of water added to the 130 µL diet provided for each larva in the artificial brood chamber. Unlike for the honey bee larvae, the amount of diet provided for the stingless bee is enough for them to complete their development without adding more diet. As each larva ingests the entire quantity of food provided, the full dose of ingested imidacloprid was known. The rearing methodology for *M. quadrifasciata anthidioides* reported here was adapted from Siqueira et al. [53].

### Survival, Body Mass and Developmental Time

The survival of individual stingless bee larvae was monitored daily in each rearing chamber throughout development, from the egg stage until adult emergence. The artificial rearing cells were opened daily and inspected for this purpose; dead individuals were identified by the absence of spiracle movement and removed. Five replicates of 24 insects from each of the five colonies were established for each dose of imidacloprid. As no egg mortality was observed, survival curves were estimated starting at hatching.

All of the insects that survived imidacloprid exposure during the larval period were weighed on an analytical scale (Sartorius BP 210D, Göttingen, Germany) to determine fresh body mass when they reached the white-eyed pupa stage (three to four days after the start of pupal period). The developmental time (days) from egg-hatching until adult emergence was also recorded for each insect. Worker body size was not determined since worker bees within a colony are monomorphic with very little variation in body size. Besides, fresh body mass was determined and it is a surrogate measure of body size.

### Walking Behavior

Surviving young adult workers fed on an imidacloprid-contaminated diet were subjected to behavioral walking bioassays. Each insect was individually transferred to an arena comprising a Petri dish (9 cm in diameter and 2 cm high) lined at the bottom with filter paper (Whatman no. 1) and along the inner walls with Teflon® PTFE (DuPont, Wilmington, DE) to prevent escape. The movement of each insect within the arena was recorded for 10 min and digitally transferred to a computer using an automated video tracking system equipped with a CCD camera (ViewPoint Life Sciences Inc., Montreal, Canada). The parameters recorded in each arena were walked distance (cm), velocity (cm/s), resting time (s), and number of stops within the arena. Walking behavior was recorded for each adult insect one, four and eight days after emergence, before the young adult workers were able to fly [54]. Behavioral bioassays were carried out between 14∶00 and 18∶00 h in a room with artificial incandescent light and an average temperature of 25±3°C. Bioassays were carried out for nine doses of imidacloprid (including a water-only control), three adult ages (one, four and eight days after emergence) and five replicates, corresponding to an average of five individual bees from each colony.

### Morphometry of Mushroom Bodies

The mushroom bodies of young adult worker bees exposed to an imidacloprid-contaminated or control diet were subjected to morphometric analysis. These individuals were collected at the same ages from the same colonies as used for the behavioral bioassays and first subjected to the walking regime described above. Brains were individually dissected in insect physiological solution (0.1 M phosphate buffer at pH 7.4) and fixed in 4% paraformaldehyde in 0.1 M phosphate buffer (pH 7.4) for 24 h at 4°C. The fixed samples were rinsed in phosphate buffer, dehydrated in an ethanol series (70%–100%), and embedded for 24 h in JB-4 historesin without hardener. After this period, the samples were placed into JB-4 historesin with hardener following the manufacturer’s recommendations for use on a microtome.

**Figure 1:**
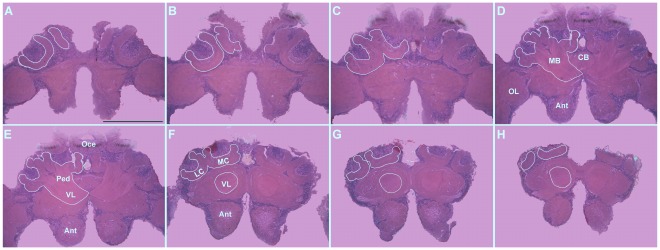
Serial histological sections of the brain of a stingless bee worker (*Melipona quadrifasciata anthidioides*). The edges of the mushroom bodies are delineated with white lines. The sections are ordered such that A, D and H represent the beginning, middle and end of the structure, respectively. MC, median calyx; LC, lateral calyx; VL, vertical lobe; MB, mushroom bodies; Oce, oceli; Ant, antennal lobe; OL, optic lobe; CB, central body. Bar: 500 µm.

Five brains were used for each combination of imidacloprid dose and age (one, four and eight days after emergence). Each brain was serially sectioned into 7 µm-thick slices with a glass knife on an automatic microtome. The sections were stained with hematoxylin and eosin and subsequently photographed using a digital camera (Canon Power Shot A640, Lake Success, NY, USA) coupled to a light microscope (Axioskop 40, Zeiss, Göttingen, Germany). One of the first six sections in which the mushroom bodies were apparent was randomly selected for area measurement (µm^2^). The same measurement was performed at each of six section intervals with the software Image-Pro Plus™ (MediaCybernetics, Bethesda, MD, USA). The volume of the mushroom bodies was determined by measuring the medial lobe, vertical lobe, peduncle and lateral and medial calyxes (Fig. 1), applying the Cavalieri method [55]. The volume estimated with this method differs by less than 5% from the volume estimated using all of the sections through this structure [37], [56], [57].

### Statistical Analyses

The results of the mortality bioassays were subjected to survival analysis using the non-parametric procedure LIFETEST from SAS [58], in which survival curves are obtained using Kaplan-Meyer estimators. The bees that survived through the eighth day after adult emergence were treated as censored data, and the median survival times (LT_50_s) for bees exposed to each imidacloprid dose were subsequently subjected to regression analysis with insecticide dose as the independent variable, using the REG procedure in SAS [58]. Insect body mass and developmental time were also subjected to regression analysis with imidacloprid dose as the independent variable (REG procedure from SAS) [58].

Mushroom body volume data were subjected to an analysis of covariance with adult age as the independent variable and imidacloprid dose as the covariate (GLM procedure in SAS). This analysis was complemented by linear regression analyses (REG procedure in SAS) with imidacloprid dose as the independent variable for each adult age considered [58]. The walking behavior data were not subjected to analyses of covariance because the results at different adult ages were not independent from each other. Therefore, linear regression analyses were carried out for each individual behavioral trait using imidacloprid as the independent variable for each adult age (REG procedure in SAS) [58]. The assumptions of normality and homoscedasticity were checked before data analysis (UNIVARIATE procedure in SAS) [58].

## Results

### Survival, Body Mass and Developmental Time

**Figure 2:**
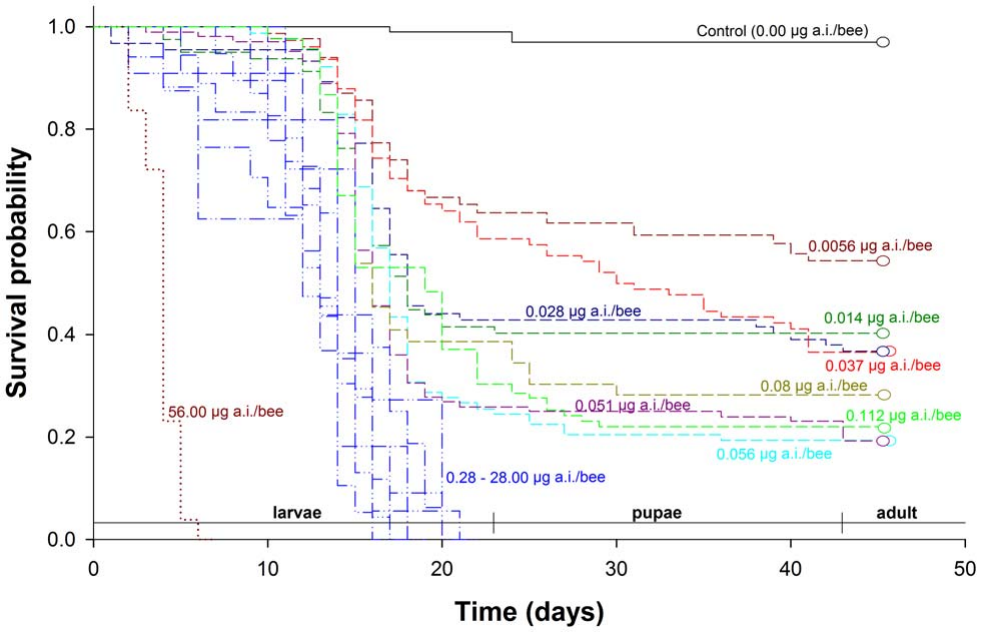
Survival plots of stingless bee workers (*Melipona quadrifasciata anthidioides*) reared on imidacloprid-contaminated diets containing increasing doses of the insecticide. The survival curves of workers bees exposed to imidaclorpid doses between 0.28 e 28 µg a.i./bee were not significantly different and were therefore coded with the same color (i.e., blue).

**Figure 3:**
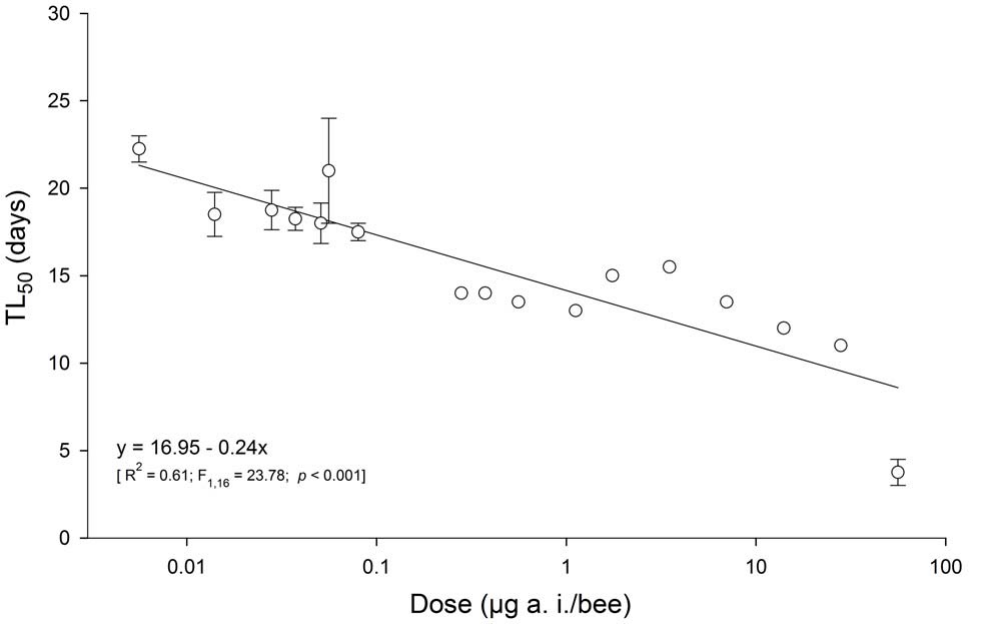
Median survival times (TL_50_) of stingless bee workers (*Melipona quadrifasciata anthidioides*) reared on imidacloprid-contaminated diets containing increasing doses of the insecticide.

Survival of stingless bee larvae exposed to imidacloprid was significantly impaired (survival curves obtained using Kaplan-Meier estimators; Log-rank test: χ^2^ = 136.13, d.f. = 17, *p*<0.001) (Fig. 2). The survival curves at doses between 0.28 e 28 µg a.i./bee were similar (*p*>0.05) and all of the worker larvae exposed to doses within this range died before reaching the pupa stage (Fig. 2). An even stronger effect of imidacloprid was observed at 56.00 µg a.i./bee, where the larvae usually survive for less than five days. Survival rates were above 50% only at the lowest imidacloprid dose used (0.0056 µg a.i./bee) and among the control (97% survival), with a negative correlation between the insecticide dose and the median survival time (TL_50_) (Fig. 3). In contrast, exposure of larvae to imidacloprid did not significantly affect developmental time (average results of pooled data was 41.09±2.48 days) or body mass (average results of pooled data was 82.85±2.76 mg) (F_1,273_>3.85; *p*>0.05). No diet rejection was observed in the experiment with the stingless bee larvae ingesting the whole content of diet provided for each one (i.e., 130 µL/larvae), regardless of the imidacloprid contamination.

### Morphometry of the Mushroom Bodies

**Figure 4:**
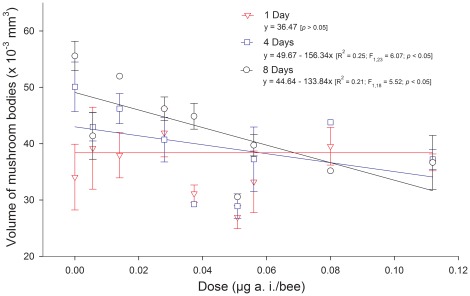
Volume of the mushroom bodies in the brains of the stingless bee workers (*Melipona quadrifasciata anthidioides*) reared on imidacloprid-contaminated diets during the larval period. The symbols represent means and standard error.

The covariance analysis of mushroom body volume indicated a significant interaction between imidacloprid dose and adult age (F_2,67_ = 3.61; *p* = 0.03). This result indicates that the effect of imidacloprid doses differs at each adult age and such differences were therefore described using individual regression analyses (Fig. 4). The simple linear models used to describe the effect of imidacloprid on the mushroom bodies were selected based on parsimony, high F values and steep increase of R^2^ with model complexity, besides of respecting the assumptions of the covariance analysis used. The mushroom bodies of newly emerged adult workers (one day old) were not significantly affected by imidacloprid, but their development was thereafter significantly impaired by imidacloprid, as reflected by the reduced volume observed in older insects. As expected, the untreated insects exhibited an increase in mushroom body volume with aging (from 34.06±5.84×10^–3^ mm^3^ for one-day-old adults to 50.10±4.40×10^–3^ mm^3^ and 55.57±2.62×10^–3^ mm^3^ for four- and eight-day-old adults). In contrast, when the insects were exposed to the insecticide during larval development, this increase was compromised, even more so at higher doses, where a 36% reduction in volume was observed under the highest dose eight days after emergence (Fig. 4).

### Walking Behavior

**Figure 5:**
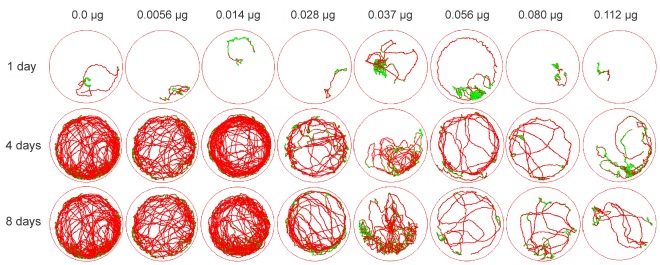
Representative tracks showing the movement (10 min) of individual adult workers (at different times following emergence) of the stingless bee *Melipona quadrifasciata anthidioides* reared on imidacloprid-contaminated diet during the larval period. Red tracks indicate high walking velocity, while green tracks indicate low (initial) velocity.

**Figure 6:**
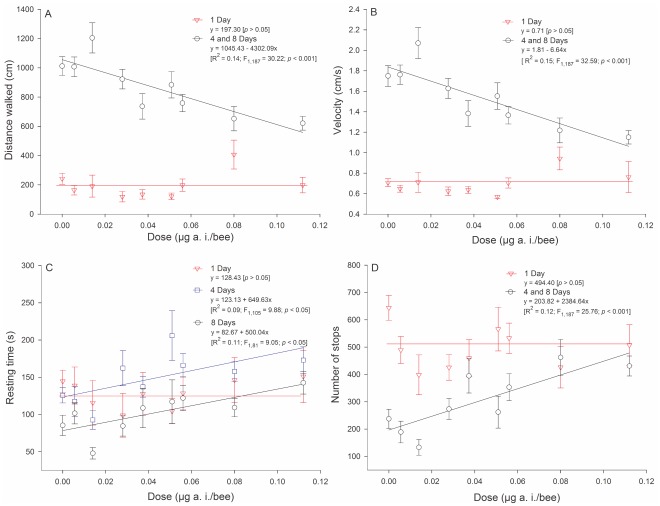
Effect of larva ingestion of imidaclorprid-contaminated diet on the distance walked (A), walking velocity (B), resting time (C) and number of stops in the arena (D) of individual adult workers (at different ages following emergence) of the stingless bee *Melipona quadrifasciata anthidioides*. The symbols represent means and standard error.

Tracks representative of the typical walking behavior of young adult workers are exhibited in Fig. 5. Larval ingestion of imidacloprid did not affect the walking behavior of one-day-old adults relative to controls, whereas older adults (four and eight days after emergence) were affected (*p*<0.05) (Fig. 6). The distance walked, walking velocity and number of stops in the arena were similar between four- and eight-day-old adults. These data were therefore pooled for the regression analysis. In addition, there was a dose-dependent overall impairment in walking activity following imidacloprid ingestion. High doses of imidacloprid led to reductions in distance walked (Fig. 6A) and walking velocity (Fig. 6B) as well as increases in resting time (Fig. 6C) and number of stops in the arena (Fig. 6D) in the four- and eight-day-old adults.

## Discussion

Pesticide application is a common agricultural practice, particularly in the tropics, despite the potential harm these compounds pose to non-target species [47], [49]. Pesticide residues can accumulate and persist in honey bee hives [15]. Some of these residues derive from pesticide applications targeting *Varroa* parasitic mites, but most can be attributed to exposure of bee foragers to contaminated plants by contact with contaminated surfaces in the field [59], harvesting of contaminated pollen and nectar [9], [15] or ingestion of contaminated sap from plants originating from insecticide-coated seeds [16].

Most studies on the off-target effects of pesticides on bees (mainly the honey bee) have focused on the adult stage. However, all developmental stages and castes are potentially affected by pesticide residues [39], [43], [60]–[67]. Furthermore, insecticide-induced reductions in progeny can lead to decreases in the rate of adult emergence and are likely more harmful to the colony than the direct acute mortality of adult bees during foraging [25], [60]. In the present study, we successfully reared workers of the stingless bee (*M. quadrifasciata anthidioides*) under controlled conditions and assessed the lethal and sublethal effects of the neonicotinoid insecticide imidacloprid on larvae exposed to a contaminated diet.

We report that chronic ingestion of imidacloprid by stingless bee larvae of *M. quadrifasciata anthidioides* results in high toxicity, with only 55% survival at the lowest dose tested (0.0056 µg a.i./bee). Doses higher than 0.28 µg a.i./bee prevented the larvae from even reaching the pupa stage. Doses lower than 0.0056 µg a.i./bee may also have significant lethal and sublethal consequences in this species but are unlikely to delay insect development or cause pupa or adult malformations, as previously reported in honey bees exposed to several other insecticides, particularly insect growth regulators [44], [64], [65]. Such an effect on development is expected for growth regulators but not for neurotoxic compounds such as imidacloprid. However, imidacloprid compromised the development of the mushroom bodies in the brains of young adult *M. quadrifasciata anthidioides* workers and impaired their walking activity.

Mushroom bodies are the primary structures responsible for the processing and integration of multisensory information, memory and learning in insects [68]–[73]. Neuroblasts, which are neuronal precursor cells, give rise to the intrinsic neurons that form the mushroom bodies in the brain during the pupa stage in the honey bee [38], [74], [75]. Neuroblasts are not present in adults, preventing them from forming new neurons [38], [74], [75]; however, bee mushroom bodies exhibit plasticity after adult emergence. This plasticity is associated with age and experience and results in an increase in volume with aging [37], [38], [76]. We observed that the mushroom bodies of the stingless bee *M. quadrifasciata anthidioides* also increase in volume with aging but that imidacloprid ingestion during larval development compromised this increase. The effect was more drastic with higher insecticide doses. Exposure to hydroxyurea also led to a reduction in mushroom body size in adult honey bees [77]; however, this effect seems to be distinct from that of imidacloprid in the stingless bee because while hydroxyurea treatment led to neuroblast death, imidacloprid did not interfere with mushroom body formation but rather with age-dependent plasticity, particularly at doses ≥0.08 µg a.i./bee. It remains unclear whether imidacloprid can kill neuroblasts of *M. quadrifasciata anthidioides*.

Imidacloprid is reported to interfere with the mushroom body calyxes of adult honey bees, where it binds to nicotinic acetylcholine receptors (nAChR) on the intrinsic neurons [34], [78], [79]. Cholinergic synaptic transmissions are consequently blocked, and cellular metabolism is altered, causing memory problems related to sensorial and motor systems [26], [27], [29], [80], [81]. Stingless bees exposed to imidacloprid during the larval stage presented adult-onset effects related to the age-dependent development of their mushroom bodies and consequent effects on walking activity and behavior. The impairment in walking behavior induced by imidacloprid is likely a consequence of inhibited mushroom body development because these structures integrate multimodal signals from different neural systems, including motor control [82]. Stingless bee larvae exposed to imidacloprid may also present memory problems related to the impairment in mushroom body development, as reported for honey bees [26], [27], [29].

The adverse effects of imidacloprid were not apparent in newly emerged adult stingless bees, but altered walking behavior was observed after four days of emergence. This result is not surprising because, as in honey bees [83], newly emerged adults are not particularly active. Their activity increases with age, eventually resulting in flight and foraging [54]. It seems that the impairment in walking behavior is likely a consequence of the effect of imidacloprid on the mushroom bodies. However, such a consequence may be indirect since the mushroom bodies are primarily associated with insect learning and memory (although they are also involved in the processing and integration of multisensory information), which are associated with the insect motor activities. In addition, imidacloprid may have direct effect in the motor neurons inducing their slow depolarization, as reported in cockroaches [84], directly compromising the insect motor activity, what deserves attention. If the walking behavior of young adult stingless bees is compromised, it is probable that their subsequent flight and foraging behaviors would be even more impaired.

Young adults of the stingless bee *M. quadrifasciata anthidioides* carry out distinct tasks within the hive, including detritus removal, comb production, food storing and larvae feeding [54]. Impairment of walking activity during this stage is likely to compromise all of these activities. More complex behaviors carried out by bees, including foraging at later adult ages, are closely dependent on memory, learning and motor ability [35], [85]. Therefore, even if the imidacloprid-exposed bees were to reach foraging age, they may be unable to perform functions that demand a high level of integrity in the brain regions affected by the insecticide. As colonies of stingless bees are not as populous as those of honey bees, pesticide impacts, e.g., direct mortality and sublethal effects compromising crucial tasks, are likely to have more serious consequences on the fitness of the colony, compromising its structure, organization and survival [48], [66], [67]. Native species of stingless bees should therefore be targeted in pesticide impact studies, not only because of their economic and ecological importance, but also because of their potentially higher vulnerability to these compounds.
